# Radiovaccination Hypothesis

**DOI:** 10.7759/cureus.1135

**Published:** 2017-04-04

**Authors:** Libni Eapen

**Affiliations:** 1 Radiation Medicine Program, The Ottawa Hospital Cancer Centre

**Keywords:** melanoma, radiovaccination, radiotherapy

## Abstract

The details of patients who have entered remission from metastatic melanoma following palliative radiotherapy are reported. We review the relevant immune physiology and radiotherapy particulars and propose the hypothesis that radiovaccination with high fractional dose to skin metastases can stimulate the development of a robust systemic anti-tumoral immune response capable of causing remission of metastatic disease.

## Introduction

Spontaneous remissions in cancer are well documented and attributed to immune responses [1-2]. Radiotherapy with or without co-immunomodulation can trigger tumor shrinkage or clearance well beyond the irradiated portals [3]. We report four cases of metastatic melanoma entering remission with palliative radiotherapy and hypothesize that radiovaccination achieved this.

## Case presentation

### Case 1

**Figure 1:**
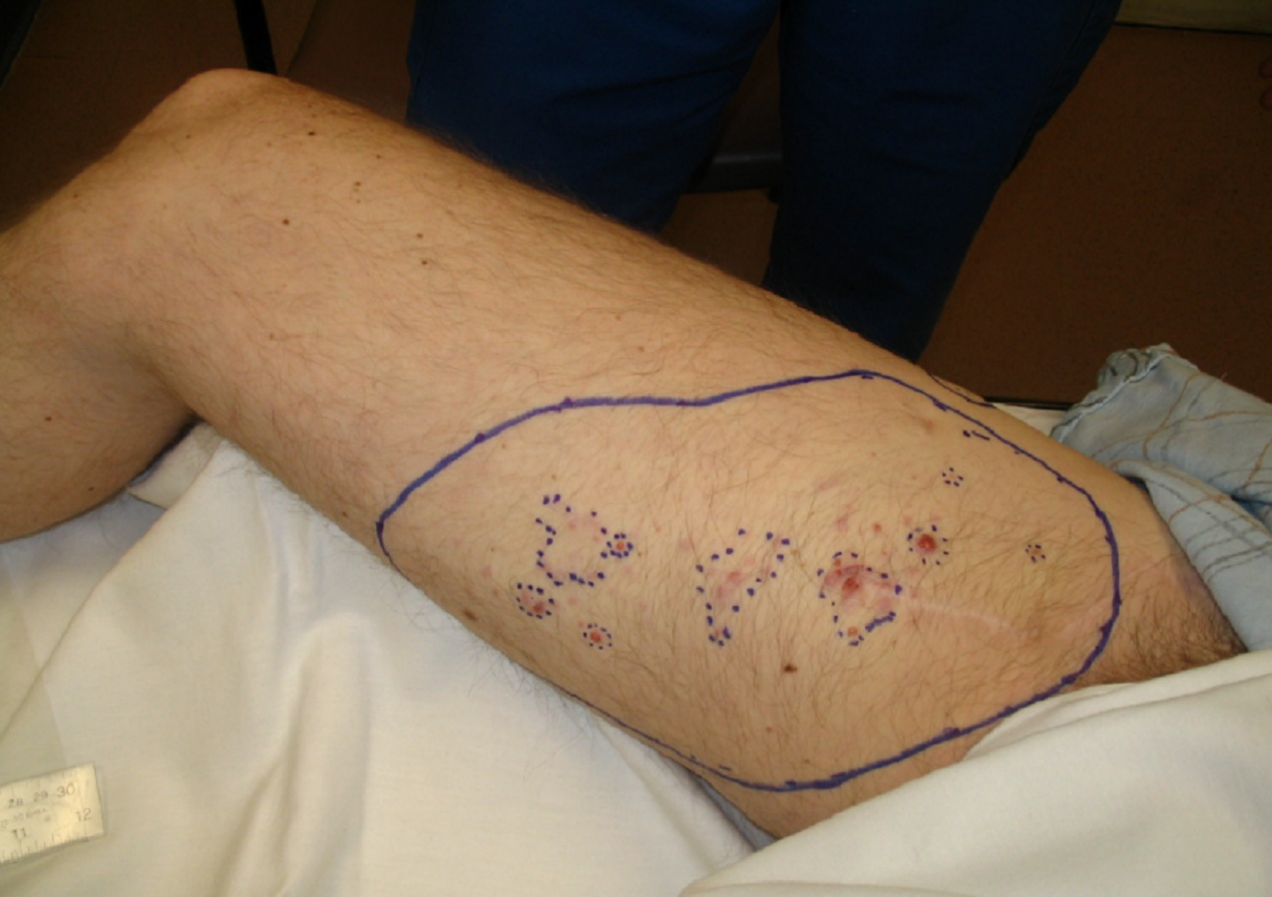
pT2bN1 M0 melanoma right lower leg

A 46-year-old man presented in February 2007 with pT2bN1 M0 melanoma of the right lower leg (Figure 1) treated with excision, sentinel node sampling, inguinal dissection, and adjuvant interferon. In April 2008, he developed upper thigh in transit metastases. He underwent radiotherapy (40 Gy/10 fractions) producing transient erythema and desquamation. All skin metastases resolved. In December 2008, he developed a chest wall and lung metastases. Tetanus booster was given anticipating chemotherapy. However, no further systemic treatment was given. All metastases resolved and he remains in remission last having been seen in 2016.

### Case 2

**Figure 2:**
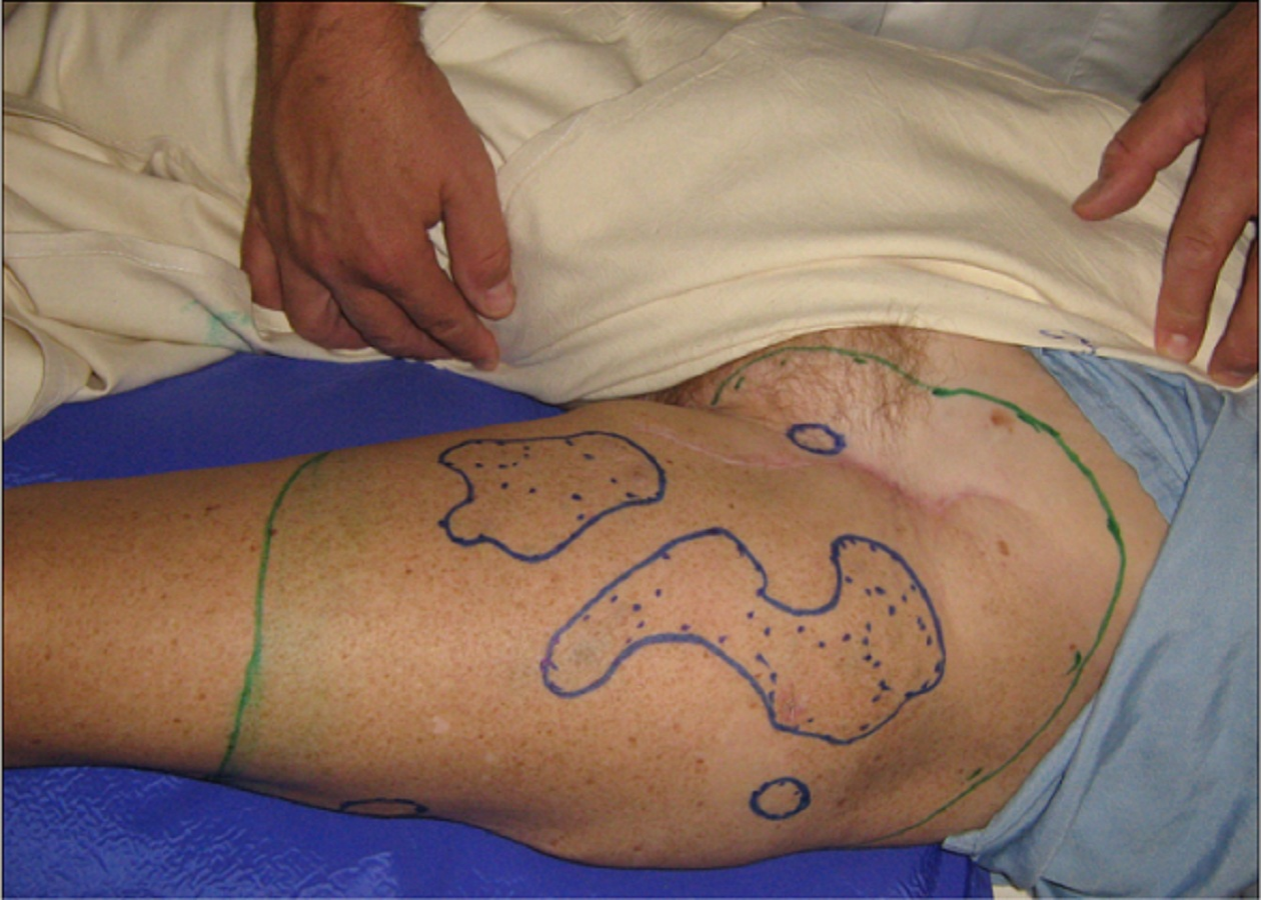
pT4N1M0 melanoma left thigh

A 51-year-old woman presented in December 2007 with pT4N1M0 melanoma of the left thigh (Figure 2) treated by excision, sentinel node sampling, and node dissection. In August 2008, she developed in transit metastases in the upper thigh. Computed tomography (CT) showed lung metastases, mediastinal and pelvic adenopathy. She underwent radiotherapy (40 Gy/10) producing erythema and desquamation. All cutaneous disease resolved and repeat imaging showed resolution of pulmonary metastases and shrinkage of adenopathy. In July 2009, she developed brain metastases treated with radiotherapy. No systemic therapy was administered and she remains in remission with the resolution of brain metastases and subcentimetric retroperitoneal nodes on imaging last having been seen in 2016.

### Case 3

**Figure 3:**
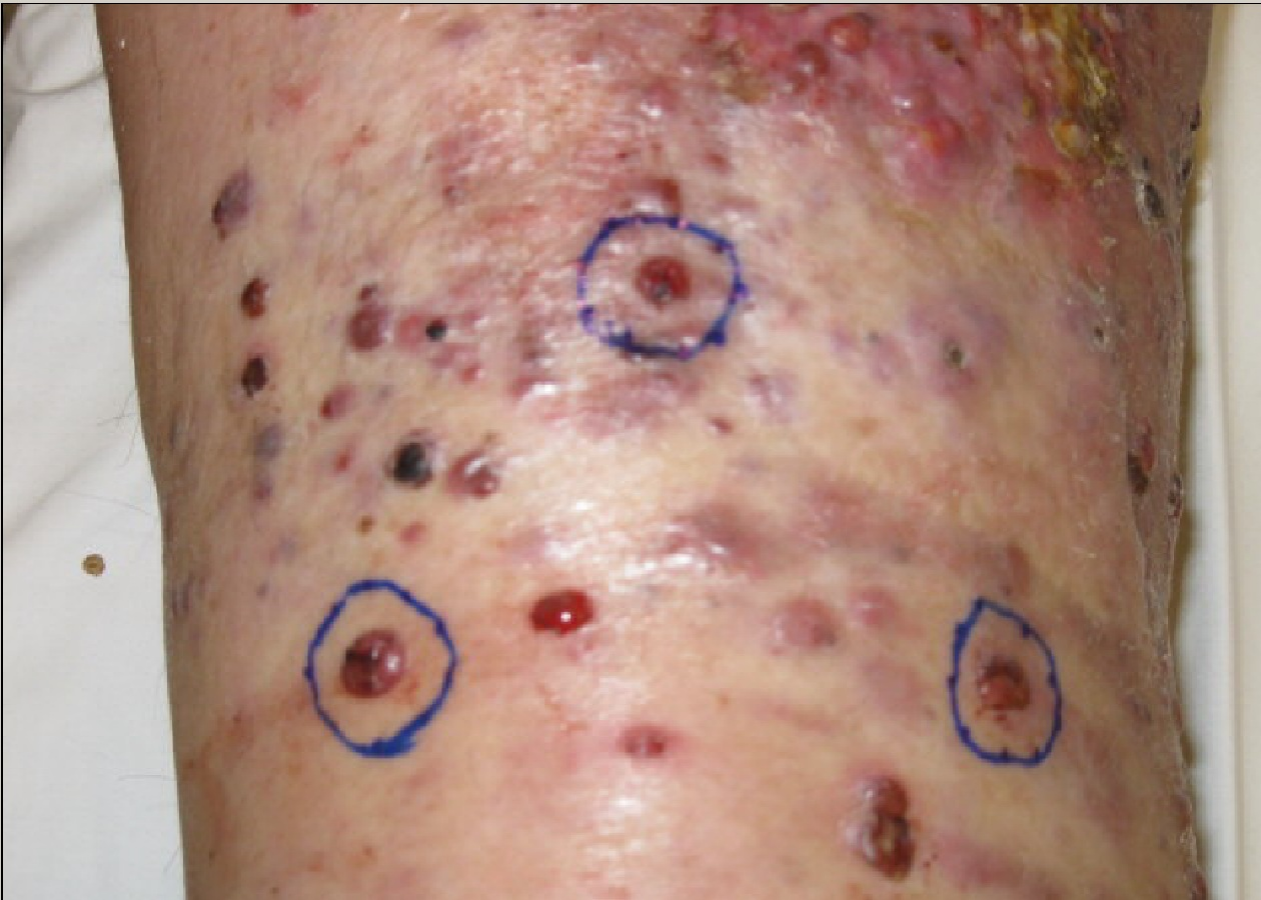
pT4 N2a M0 melanoma right foot

A 64-year-old female presented with pT4 N2a M0 melanoma on her right foot (Figure 3) in June 2008. She was treated with resection, sentinel node biopsy, node dissection, and induction interferon. In June 2009, she developed right leg in transit metastases and underwent limb perfusion with Melphalan and Actinomycin D. Her disease progressed in skin, and pelvic nodes. In April 2010, she received radiotherapy: right upper thigh (18 Gy/1), right anterior shin and right calf (30 Gy/5). In May 2010, she had four more leg lesions irradiated (20 Gy/1). She additionally commenced herbal/acupuncture therapy. By July 2011, all disease had resolved except an upper thigh tumor which was irradiated (35 Gy/5). No further systemic treatment was administered. She remained in remission when last seen in 2013.

### Case 4

**Figure 4:**
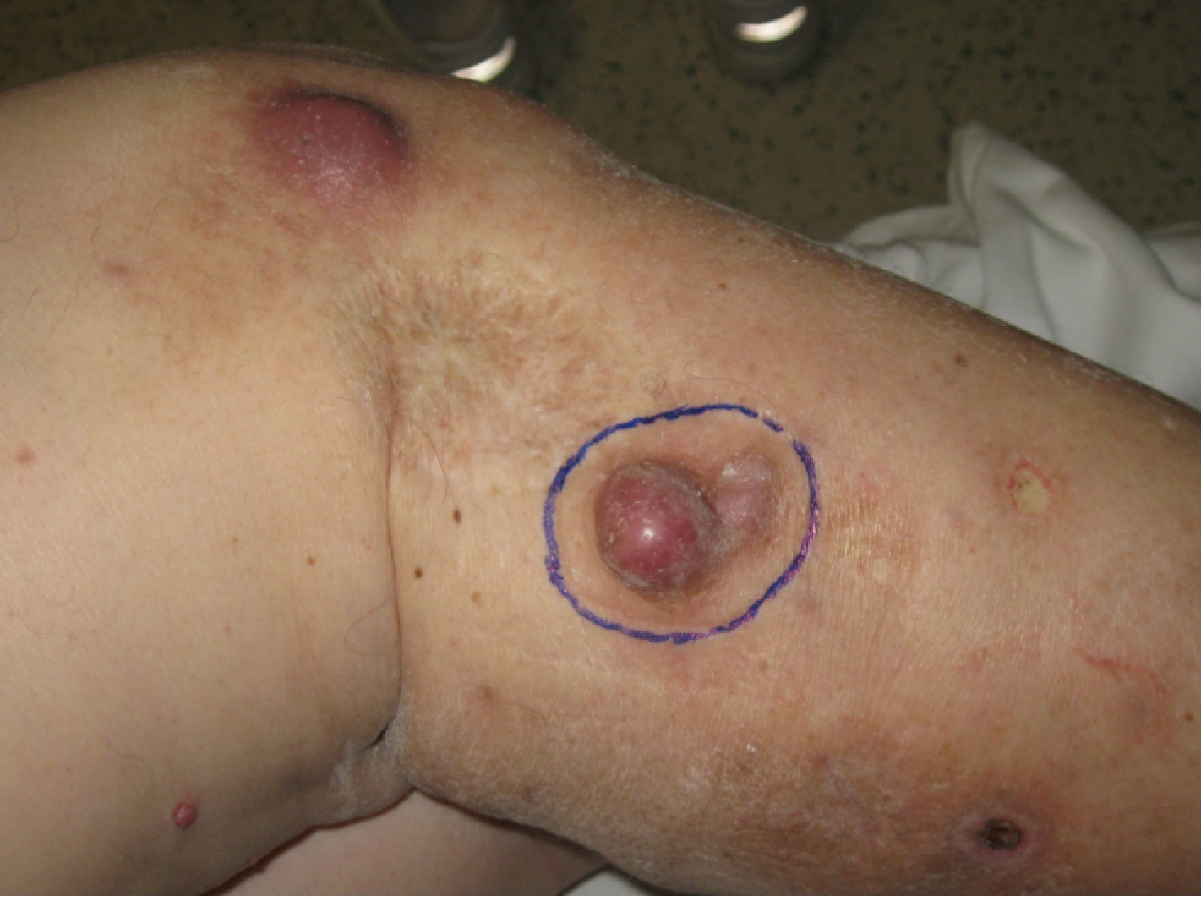
T3N0M0 right knee melanoma

A 74-year-old lady presented with T3N0M0 right knee melanoma (Figure 4) treated with resection and sentinel node sampling. In February 2008, she developed skin metastases treated with radiotherapy (20 Gy/1) to right knee and right calf and 32.5 Gy/5 to right ankle. In April 2008, three other right calf nodules were irradiated (20 Gy/1). The irradiated lesions regressed completely. In February 2009, she developed extensive in transit metastases throughout her right leg. She was started on a clinical trial of intralesional Vical in June 2009. Her best clinical response between June 2009 and April 2010 during this therapy was stability with many lesions growing but less than 25% increase. In April 2010, imaging showed lung, liver, and pelvic nodal metastases and Vical was stopped. She received radiotherapy to her right knee (37.5Gy/10). She remains in remission with one palpable leg nodule representing either tumor or fibrosis. She was last seen in 2016.

## Discussion

In 2016, there is abundant enthusiasm and optimism that patients can finally realize material gains from novel immunotherapeutic strategies. In addition to the already demonstrated benefits of CTLA-4 and programmed death ligand therapies, considerable efforts are being marshaled to explore the potential in using radiotherapy to mobilize a systemic immune-mediated tumoral response. The cases we describe suggest an exploitable variation on this theme. The radiotherapy in these patients used large fractional dose and caused skin injury. Initiating the response by primary effector cells in lymph nodes, dendritic cells link the innate and adaptive immune arms. Epidermal dendritic cells (Langerhans) [4], upon stimulation, process dying tumor cell antigens and migrate through lymphatics to nodes [5]. Dendritic cell engagement is facilitated by skin injury and augmented by exposure to inflammatory cytokines and antigens released from dying tumor cells [6-8]. This immunostimulatory milieu occurs more readily with necrotic cell death and vascular disruption that follows large fraction radiotherapy than with the relatively immune-inert process of apoptosis following conventionally fractionated radiation. Ronchese, et al. [9] demonstrated higher concentrations of dendritic cells in lymph nodes following subcutaneous versus intravenous administration of bone marrow-derived dendritic cells. It is probable that recruitment of dendritic cells from their cutaneous depot is the more efficient mechanism to maximize nodal processing. In the lymph nodes, these stimulated dendritic cells present tumor antigen-MHC1 surface complexes to T lymphocytes that initiate elaboration of a regionally generated systemically disseminated immune response.

With Stamell’s [2] case of regression of unirradiated in transit metastases following 24 Gy/3 in scalp melanoma, these are five patients with durable remissions following high fractional dose radiotherapy and significant skin injury around the tumor. The demonstrated radioresistance of cutaneous dendritic cells [10] enables them to function despite irradiation. This amalgam of enabling conditions is plausible within the following physiological paradigm. The epidermal location of tumor irradiated with large doses per fraction wreaks cellular and cytokine havoc in tumor, tumor vasculature, and enshrouding skin galvanizing dendritic cells to ferry warnings to the closest vanguard of nodal T lymphocytes. As with infectious disease vaccine prophylaxis, pertinent issues include frequency of vaccination (repeated irradiation of different tumors in the same patient to present serial challenging of nodes by tumor antigen) and dose of tumor antigen inoculum (how large a volume of tumor to irradiate).

## Conclusions

This hypothesis that radiovaccination using large fraction irradiation of skin metastases can produce a systemic antitumoral response can be tested in animals and patients alike by comparing post radiotherapy immune serology and abscopal tumor response rates following a) conventional versus high dose per fraction irradiation and b) tumoral irradiation with and without significant skin radiation dose.

Adding four cases to the one already reported in the literature merely expands the anecdote pool. However, as these four cases are from the melanoma practice at one centre within the past two years, it seems warranted to have sought a common theme rather than ascribing the outcomes to coincidence. Anecdote was the term originally used to describe the unpublished memoirs of the emperor Justinian. Its use hinted at the revelation of court secrets and intrigue. In this sense, a fuller critical examination of the therapeutic possibilities offered by this assembly of anecdotes may well reveal insights that can enable the successful conscription, by judicious radiotherapy directed at tumor AND dermis, of a patient’s own immune system towards systemic tumor eradication.
